# Effects of Huanglian-Renshen-Decoction, a Fixed Mixture of Traditional Chinese Medicine, on the Improvement of Glucose Metabolism by Maintenance of Pancreatic *β* Cell Identity in db/db Mice

**DOI:** 10.1155/2019/1232913

**Published:** 2019-03-19

**Authors:** Fen Yuan, Dingkun Wang, Leyi Ma, Xin Qin, Jing Gong, Meilin Hu, Hao Su, Hui Dong, Fuer Lu

**Affiliations:** Institute of Integrated Traditional Chinese and Western Medicine, Tongji Hospital, Tongji Medical College, HuaZhong University of Science and Technology, Wuhan, Hubei 430030, China

## Abstract

Huanglian-Renshen-Decoction (HRD) is widely used to treat type 2 diabetes mellitus (T2DM) in China. However, the underlying mechanism is unclear. We aimed to investigate the mechanism by which HRD regulates the glucose level. Forty 7-8-week-old db/db (BSK) mice were randomly assigned to the following four groups: model, low dose HRD (LHRD), high dose HRD (HHRD), and saxagliptin (SAX). Additionally, 10 db/m mice were assigned to control group. The experimental mice were administered 3.03g/kg/d and 6.06g/kg/d of HRD in the LHRD and HHRD groups, respectively, and 10mg/kg/d saxagliptin in the SAX group for 8 weeks. The control and model groups were supplied with distilled water. After the intervention, the pancreas and blood were collected and tested. Compared with that of model group, the fasting blood glucose (FBG) was significantly decreased in all intervention groups (*p *< 0.05 or 0.01), whereas fasting serum insulin (FINS) was increased significantly in both HHRD and SAX groups. The immunofluorescence images showed that the mass of insulin^+^ cells was increased and that of glucagon^+^ cells was reduced obviously in experimental groups compared to those of the model group. In addition, the coexpression of insulin, glucagon, and PDX1 was decreased in HHRD group, and the level of caspase 12 in islet was decreased significantly in all intervention groups. However, little difference was found in the number and morphology of islet, and the expression of ki67, bcl2, bax, caspase 3, and cleaved-caspase 3 in the pancreas among groups. Interestingly, the cleaved-Notch1 level was increased and the Ngn3 level in islet was decreased significantly in HHRD group. The HRD showed dose-dependent effects on glucose metabolism improvement through maintenance of *β* cell identity via a mechanism that might involve the Notch1/Ngn3 signal pathway in db/db mice.

## 1. Introduction

Type 2 diabetes mellitus (T2DM) is characterized by relative or absolute insulin deficiency that leads to hyperglyceamia [1]. A total of 347 million individuals had T2DM in 2013, and this number is predicted to climb to 400 million worldwide in 2030 [2, 3]. Obviously, T2DM has become a global health problem. However, its underlying mechanism is far from clear. A recent study showed that dedifferentiation of islet *β* cells was responsible for their failure [4]. This finding may unravel a different insight into exploration of the mechanism and provide an important potential therapeutic target for T2DM [5]. In addition, the Notch signalling pathway, which is essential for regulating pancreatic development, plays an important role in diabetes [6]. Indeed, Huang et al. demonstrated that islet regeneration was probably related to the PDX1/Notch1/Ngn3 signalling pathway [7].

The Huanglian-Renshen-Decoction consists of Coptidis Rhizoma and Panax ginseng in a ratio 1:1. Coptidis Rhizoma has shown hypoglycaemia, hypolipid, anti-inflammatory functions [8–11], and its bioactive components have been studied broadly, especially berberine. Berberine can improve glucose metabolism by benefiting the insulin resistance and GLP-1 secretion [12]. In addition, it can attenuate the impaired barrier of the intestinal mucosa to lower FBG in streptozotocin-induced type 2 diabetic rats [13]. Panax ginseng functions well as a tonifying Qi herbal medicine in the theory of Traditional Chinese Medicine. Eight active ginsenosides have been identified (Rg1, Re, Rb1, Rc, Rg2, Rb2, Rb3, and Rd) [14]. Recently, Rb1 has been shown to ameliorate glucose metabolism via activating GLP-1 secretion [15]. Moreover, Rg1 can regulate the blood glucose level by abrogating the glucose absorption in the small intestine and ameliorating gluconeogenesis in the liver [16, 17]. Compound K, which is a bioactive ingredient, has also shown an effect on decreasing apoptosis via inhibiting the AMPK/JNK signal pathway [18].

Taken together, the bioactive ingredients in both Coptidis Rhizoma and Panax ginseng showed positive effects on glucose metabolism through different mechanisms. However, few studies have investigated the antihyperglycaemic effects of HRD that occur through maintenance of the identity of pancreatic *β* cell and the underlying mechanism. In Traditional Chinese Medicine theory, T2DM is named “Xiao Ke” and is characterized by Yin deficiency and heat manifestation. Diabetic patients with Yin deficiency would be inclined to suffer *β* cell failure and decreased insulin secretion [19]. Coptidis Rhizoma functions as a heat-clearing agent and Panax ginseng plays an important role in nourishing Qi and generating Yin. Consequently, HRD performs well in clearing heat and supplying Yin. We posited that HRD could improve glucose metabolism by maintaining *β* cell identity.

## 2. Materials and Methods

### 2.1. Preparation of Drug Solutions and Intervention Dose

HRD is composed of Coptidis Rhizoma and Panax ginseng concentrated granules, which were produced by China Resources Sanjiu Medical and Pharmaceutical Co., Ltd. The mixture ratio was 1:1 (w/w). Saxagliptin was produced by AstraZeneca Pharmaceuticals LP. All drugs were purchased from Tongji Hospital Pharmacy (Hubei, China) and dissolved in distilled water. The HRD doses in the LHRD and HHRD groups were based on clinical experience and a transforming formula= (daily dose of Huanglian-Renshen decoction in humans (g))/human weight (kg)) ×9.1. (The usual human weight is 60kg in Asia and 20g/d is the daily dose of HRD in humans and the ratio of Coptidis Rhizoma and Panax ginseng is 1:1; the conversion coefficient between humans and mice is 9:1). The saxagliptin dose was based on the previous article [20].

### 2.2. Animals and Experimental Design

*Forty* 7-8-week-old male db/db (C57BL/KS-db/db) mice and 10 male db/m (C57BL/KS-db/m) mice were purchased from Nanjing Biomedical Research Institute of Nanjing University and bred in SPF circumstances in the experimental animal centre of Huazhong University of Science and Technology. After adaptation for one week, the db/db mice were allocated into four groups based on the baseline FBG and weight, and the db/m mice were assigned to the control group. All experiments were approved by the animal ethics committee of Huazhong University of Science and Technology. The drugs were administrated intragastrically. We measured the FBG, 24-hour food intake and weight weekly. After 8 weeks of intervention, all mice were anaesthetized with 50mg/kg of sodium pentobarbital by intraperitoneal injection, and blood samples were collected. The pancreas was collected under sterile conditions. The samples were fixed with 4% formaldehyde or frozen instantly in liquid nitrogen and then stored in refrigerator at -80°C. Orbital blood was collected in a sterile EP tube without heparin sodium and centrifuged at 3000rpm for 15 min at 4°C. The collected serum was stored in a refrigerator at -20°C.

### 2.3. Human Pancreas Testing

Human pancreatic sections were obtained from the Department of Pathology in Tongji Hospital. Immunohistochemistry for cleaved Notch1 and Ngn3 and immunofluorescence staining for insulin and glucagon were performed as described as in Section 2.8. All experiments were approved by the clinical ethics committee of Huazhong University of Science and Technology.

### 2.4. Qualified Analysis of the Coptisine, Palmatine, Berberine, Rg1, Rb1, and Rc in HRD by High Performance Liquid Chromatography (HPLC)

The standard substances were prepared as follows. We weighed Rg1 (5.278 mg), coptisine (0.608 mg), palmatine (0.267 mg), berberine (0.689 mg), Rb1 (5.225 mg), and Rc (5.254 mg), mixed them, and dissolved them in 25 mL methanol in a conical flask. Then, we selected and weighed 10 different batches of Coptidis Rhizoma and Panax ginseng concentrated granules. These samples were dissolved with the same volume of methanol. Finally, we prepared the HRD solution by mixing and dissolving Coptidis Rhizoma and Panax ginseng. The examination condition was as follows: chromatographic column, WATERS XTERRA ®MS C18 (4.6 mm × 250 mm, 5 *μ*m); mobile phase, acetonitrile-0.05mol/L KH2PO4 (pH value adjusted to 4 with phosphoric acid); detection wavelength, 203nm; column temperature, 30°C; flow rate, 0.9mL/min. We added 10*μ*L of the standard substance solution and different sample solutions in the column and obtained the results.

### 2.5. Glucose and Insulin Tolerance Tests

We executed tIhe PGTT and IPITT on different days. The mice were injected intraperitoneally with glucose (0.75 g/kg) after fasting for 14 h for the IPGTT and Humulin R (1 U/kg) (Eli Lilly and Co., IN, USA) after fasting for 4 h for the IPITT. The glucose levels were determined at 0, 30, 60, 90, and 120 min after injection.

### 2.6. Measurement of Blood Glucose, Insulin, and Lipids

The FBG level was detected weekly with venous blood collected from the tail using a glucose monitor (Roche, Germany) after fasting for 14h. The FINS was quantified by an ELISA kit according to the manufacturer's instructions. The serum triglyceride (TG) and total cholesterol (TC) levels were detected with a colourimetric assay (Mingdian, China). The processes were based on the product instructions.

### 2.7. Pancreatic Islet Histological Morphology

Pancreatic sections were stained with haematoxylin and eosin (H&E) to assess the histological morphology. In addition, the islet numbers and area in slide were calculated under a microscope.

### 2.8. Immunohistochemistry and Immunofluorescence

Pancreas tissues were separated, fixed, and embedded in paraffin [21]. Serial sections were deparaffinized with dimethylbenzene, and antigen retrieval was conducted using citrate buffer solution. The slides were incubated with 3% hydrogen peroxide for 15 min and then blocked with 10% goat serum for 1 hour at room temperature. The slides were incubated with primary antibodies targeting Ngn3 (AVIVA SYSTEMS BIOLOGY, OAAB15617), cleaved Notch1 (Abcam, ab8925), insulin (Abcam, ab7842), NKX6-1 (Cell Signaling Technology, #54551), glucagon (Sigma, G2654), PDX-1 (Abcam, ab47267), Ki67 (Abcam, ab15580), and caspase12 (Abcam, ab62484) at 4°C in a refrigerator overnight. For immunohistochemistry, the GTVision™+ Detection System/Mo&Rb (Gene Tech, GK6005) was used to examine the signal based on the guidelines. For immunofluorescence staining, the signal was detected using FITC-labelled IgG fluorescence.

### 2.9. Western Blotting Assay

The procedure was performed partly as described previously [22]. Briefly, the protein lysis solutions were separated through gel electrophoresis and transferred onto PVDF membranes. The membranes were incubated with primary antibodies against bcl2 (Abcam, ab182858), bax (Abcam, ab32503), caspase 3 (Cell Signaling Technology, #9662), cleaved-caspase3 (Cell Signaling Technology, #9664), *β*-tubulin (Abbkine, #A01030), and *β*-actin (Abbkine, A01010) in a 4°C refrigerator overnight. Then, the membranes were coincubated with fluorescent secondary antibodies. The Odyssey imaging system was used to analyse protein expression.

### 2.10. Statistical Analysis

Data was expressed as the mean ± SD or mean± SEM. First, all data were tested for normality using Shapiro-Wilk. When* p *> 0.05, we adopted one-way ANOVA to compare the differences among the groups. When* p *< 0.05, we performed Kruskal-Wallis analysis.* P *< 0.05 was considered a significant difference. All statistical analyses were conducted with the raphPad Prism® 5 software.

## 3. Results

### 3.1. Qualified Analysis of Coptisine, Palmatine, Berberine, Rg1, Rb1, and Rc in HRD

**Figure 1:**
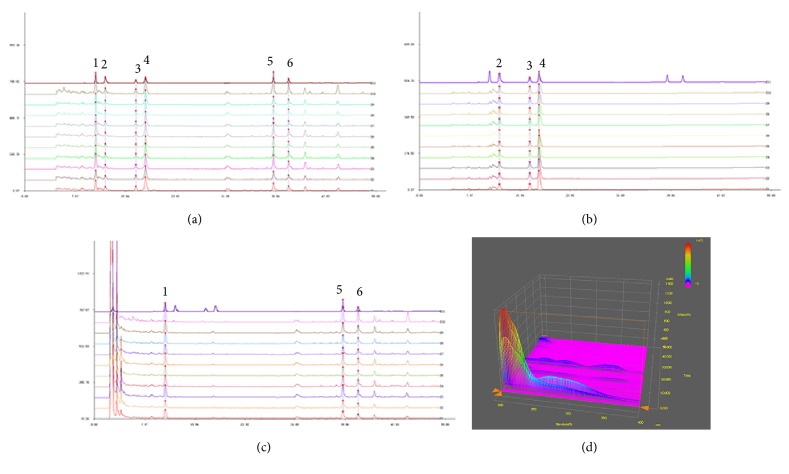
*HPLC fingerprint of the Huanglian-Renshen-Decoction.* (a) HPLC fingerprint of the standard substances showing six different peaks. (b) HPLC fingerprint of Coptidis Rhizoma with the peaks 2, 3, and 4 and (c) HPLC fingerprint of Panax ginseng with peaks 1, 5, and 6, respectively. (d) The 3D fingerprint of the Huanglian-Renshen-Decoction. (1) Rg1 (PubChem CID: 441923); (2) coptisine (PubChem CID: 72322); (3) palmatine (PubChem CID: 19009); (4) berberine (PubChem CID: 2353); (5) Rb1 (PubChem CID: 449635); and (6) Rc (PubChem CID: 12855889).

HPLC maps were obtained, as shown in Figure 1. The standard curves of the standard solutions of coptisine, palmatine, berberine, Rg1, Rb1, and Rc are shown in Figure 1(a). The curves of peaks 2, 3, and 4 shown in Figure 1(b) represent coptisine, palmatine, and berberine in Coptidis Rhizoma, respectively. The curves of peaks 1, 5, and 6 represent Rg1, Rb1, and Rc in Panax ginseng, respectively (Figure 1(c)). The 3D fingerprint is shown for the Huanglian-Renshen-Decoction (Figure 1(d)).

### 3.2. Effects of HRD on Glucose and Lipid Metabolism in db/db Mice

**Figure 2:**
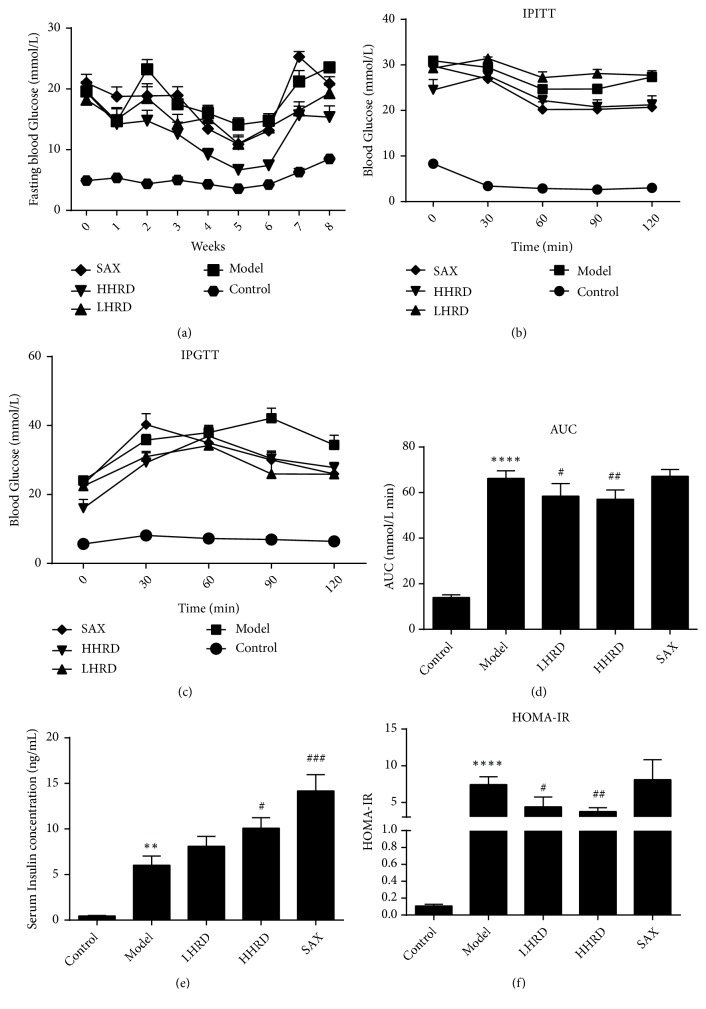
*Effects of HRD on glucose metabolism.* (a) The FBG in each group is presented as the mean ± SEM. The difference between the control and model groups was significant throughout the experiment (*p *< 0.0001);* p* < 0.05 for model versus LHRD at the second and seventh weeks;* p* < 0.05 for model versus HHRD from the second to eighth weeks; no difference was detected between the model and SAX groups. (b) An IPITT was conducted. Blood glucose level is shown as mean ±SEM. The significant gap between control and model existed (*p*< 0.0001).* p* < 0.01 for model versus HHRD at 120 min;* p* < 0.05 for model versus SAX at 60 min, 90 min, and 120 min. (c) The data are shown as the mean ± SEM.* p *< 0.0001 for control versus model at all time-points;* p* < 0.05 for model versus LHRD at 90 min and 120 min; p < 0.05 for model versus HHRD at 0 min and 90 min;* p* < 0.05 for model versus SAX at 90 min and 120 min. (d) The AUC in the IPGTT is shown as the mean ± SD. (e) The FINS is shown as the mean ± SEM. (F) The HOMA-IR is shown as the mean ± SD. n=5-8 mice in each group. *∗∗* represented* p* < 0.01 versus control group, *∗∗∗∗* represented* p* < 0.0001 versus control group, ^#^ represented* p* < 0.05 versus model group, ^##^ represented* p* < 0.01 versus model group, ^###^ represented* p* < 0.001 versus model group.

We measured the FBG levels. As shown in previous studies [23–25], HRD attenuated the glucose level (Figure 2). In comparison to that of the control group, the FBG was much higher in the model group (*p* < 0.0001). After the intervention, the FBG level was decreased in all intervention groups, especially in the HHRD group. In addition, the HHRD group exhibited elevated insulin sensitivity at 120 min in the IPITT and decreased blood glucose in the IPGTT. The FINS was increased significantly in both the HHRD and SAX groups. The area under the concentration-time curve (AUC) in the IPGTT (Figure 2(d)) and homeostasis model assessment-insulin resistance (HOMA-IR) (Figure 2(f)) were decreased in HRD-treated mice, whereas the AUC and HOMA-IR in the SAX group showed no significant differences compared to the model control. These results may indicate that insulin resistance (IR) is serious at the serious stage of diabetes and that saxagliptin has little effect on IR. Compared with those of the control group (Supplementary Figure 1), the body weights, 24-hour food intakes, and serum TG and TC levels increased significantly in the model group (p< 0.0001). However, no difference was found among the intervention groups and the model group.

### 3.3. Effects of HRD on the Islet Morphology, Quantity, and Area in the db/db Mice

**Figure 3:**
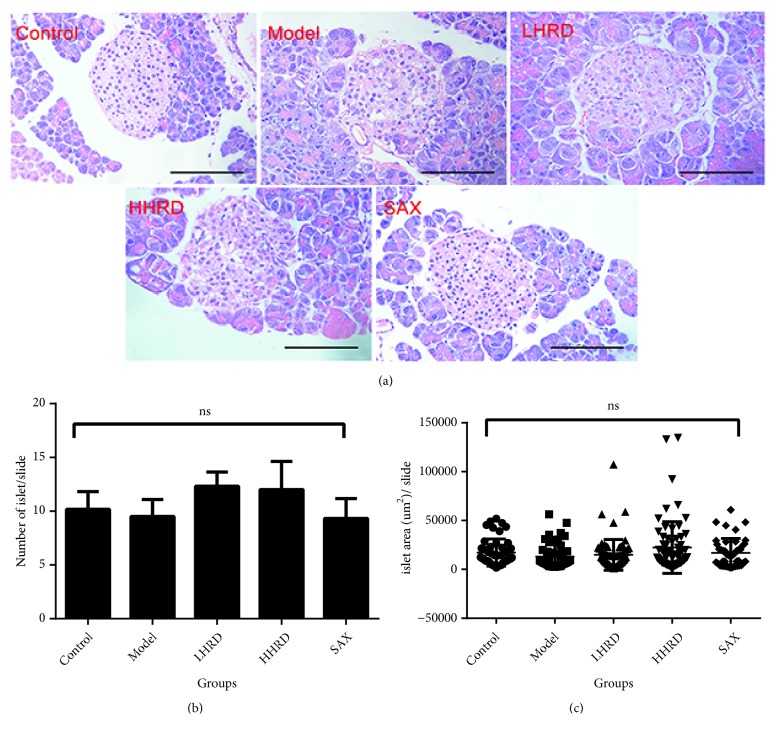
*Islet histology based on H&E staining.* The islet quantity and area were analysed by one-way ANOVA. (a) Pictures of H&E-stained sections showing the islet histology. (b) The scatter diagram presents the islet area in a slide as the mean ± SD. (c) The column shows the islet number in a slide as the mean ± SEM. No difference was found between groups. n=6-10 mice in each group. ns, not significant. All images were taken with electronic microscope (×400).

The islet morphology, quantity, and area were not different between the model and control groups (Figure 3). In addition, none of the treatment groups showed statistically significant differences compared to the model group.

### 3.4. Effects of HRD on Insulin, Glucagon, and NKX6.1 Expression in Islets

**Figure 4:**
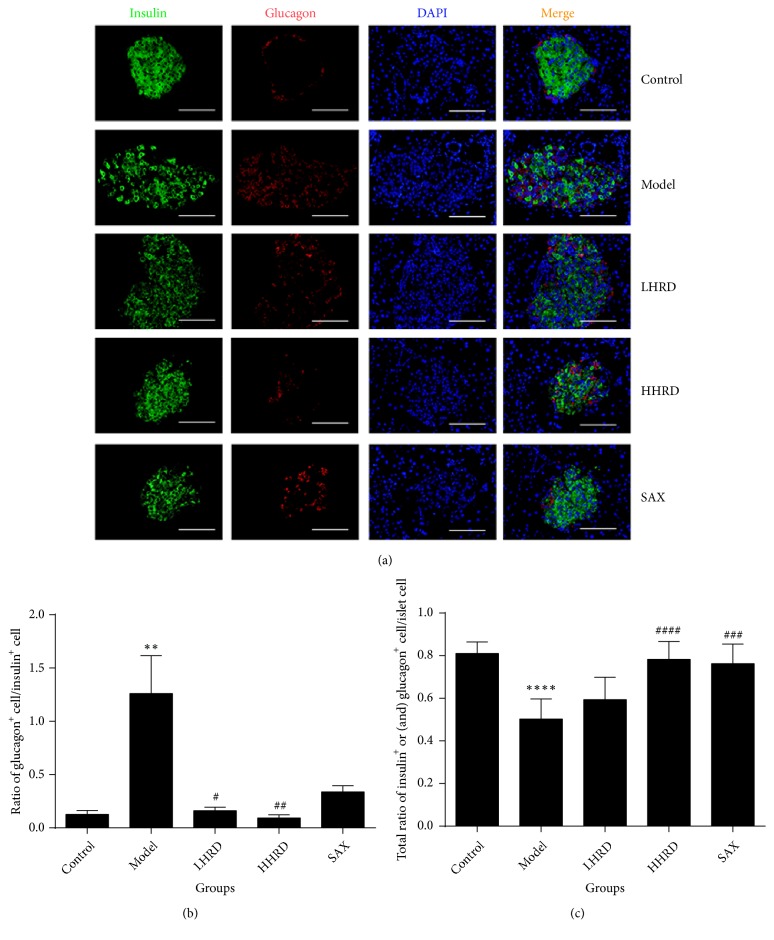
*Immunofluorescence images of insulin and glucagon in the pancreas.* (a) Immunofluorescence images showing the glucagon (red) and insulin (green) expression. DAPI staining indicates the nuclei (blue). (b) The data show the ratio of glucagon^+^ cells to insulin^+^ cells as the mean± SEM. (c) The data show the total ratio of glucagon^+^ and/or insulin^+^ cell to islet cells as the mean± SD. *∗∗* represented* p* < 0.01 model versus control group, *∗∗∗∗* represented* p* < 0.0001 model versus control group, ^#^ represented* p* < 0.05 intervention versus model group, ^##^ represented* p* < 0.01 intervention versus model group, ^###^ represented* p* < 0.001 intervention versus model group. All immunofluorescence images were taken with a fluorescence microscope (×400).

**Figure 5:**
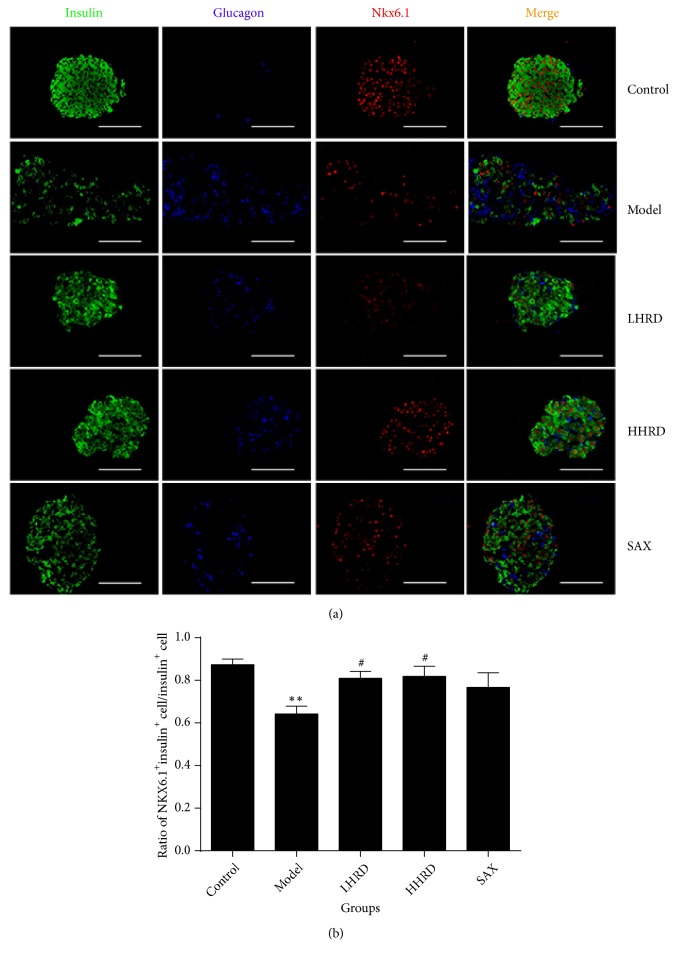
*Immunofluorescence images for NKX6.1 in the pancreas.* (a) Immunofluorescence images showing glucagon (blue), insulin (green), and NKX6.1 (red) coexpression. (b) The data show the ratio of NKX6.1^+^insulin^+^ cells to insulin^+^ cells as the mean± SEM. n= 4-8 mice in each group. *∗∗* represented* p* < 0.01 versus control group, ^#^ represented* p* < 0.05 intervention versus model group. All immunofluorescence images were taken with a fluorescence microscope (×400).

Compared with those of the control group (Figure 4), the ratio of glucagon^+^ to insulin^+^ cells was significantly increased (*p* < 0.01), whereas the total percentage of insulin^+^ and/or glucagon^+^ cells in islets was decreased significantly in the model group (*p* < 0.001). In Figure 5, the ratio of cells coexpressing insulin^+^ and NKX6.1^+^ to insulin^+^ cells was decreased significantly in the model group (*p* < 0.01). After the intervention, the ratio of glucagon^+^ cells to insulin^+^ cells was decreased (*p* < 0.05 for LHRD,* p* < 0.01 for HHRD), whereas the ratio of cells coexpressing insulin^+^ and NKX6.1^+^ to insulin^+^ cells increased significantly (*p* < 0.05 for LHRD and HHRD). The total percentage of insulin^+^ and/or glucagon^+^ cells in islets increased significantly (*p* < 0.0001 for HHRD,* p* < 0.001 for SAX). All immunofluorescence images were taken with a fluorescence microscope (×400).

### 3.5. Effects of HRD on Insulin, Glucagon, and PDX1 Coexpression in Islets

**Figure 6:**
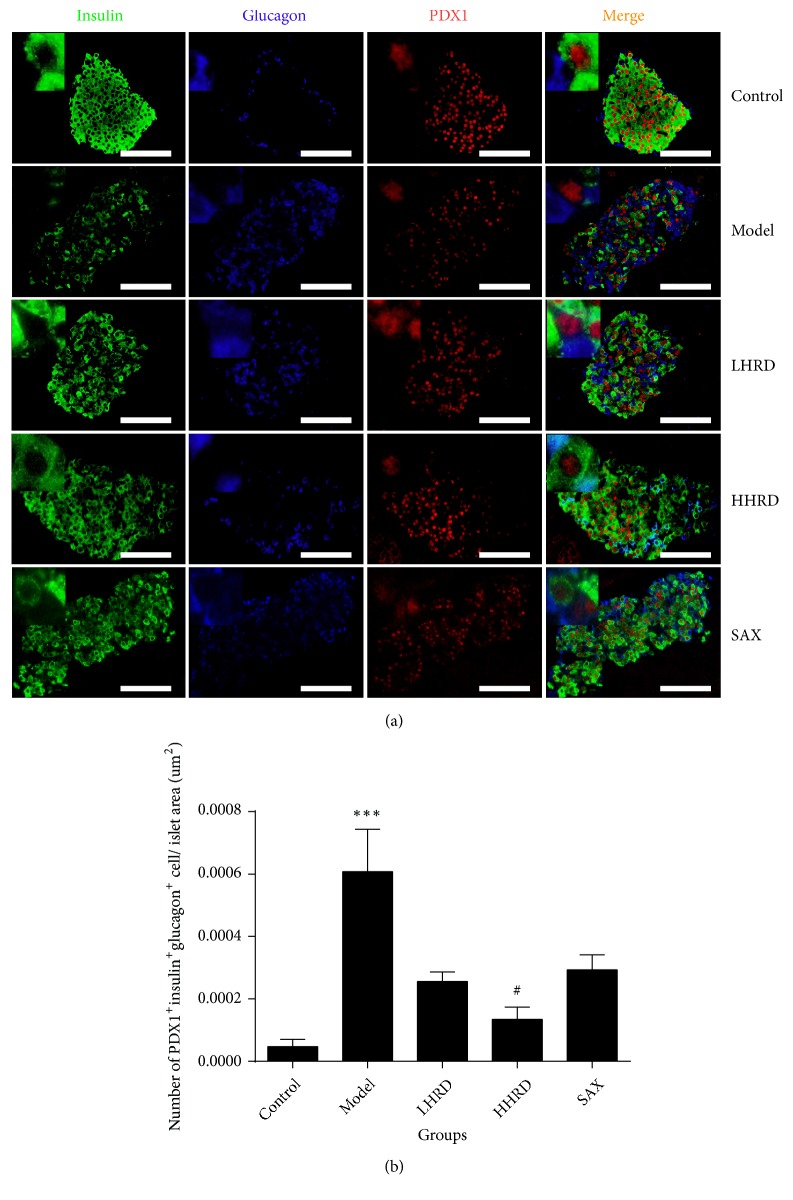
*Immunofluorescence images of PDX1/insulin/glucagon coexpression in pancreas.* (a) The immunofluorescence images show glucagon (blue), insulin (green), and PDX1 (red) coexpression in the islets. (b) The data showed the ratio of PDX1^+^ insulin^+^ glucagon^+^ cells to the islets area (um^2^) as the mean ± SEM. n= 6-8 mice in each group. *∗∗∗* represented *p* < 0.001 versus control group, ^#^ represented* p* < 0.05 versus model group. All immunofluorescence images were taken with a fluorescence microscope (×400).

Compared with that of the control group (Figure 6), the ratio of cells coexpressing PDX1, insulin, and glucagon to the islet area was increased significantly in the islets in the model group (*p *< 0.001). After the intervention, the ratio was lowered significantly in the HHRD group (*p* < 0.05). However, the difference was not significant in the LHRD and SAX groups.

### 3.6. Effects of HRD on Pancreatic Cells Proliferation and Apoptosis

**Figure 7:**
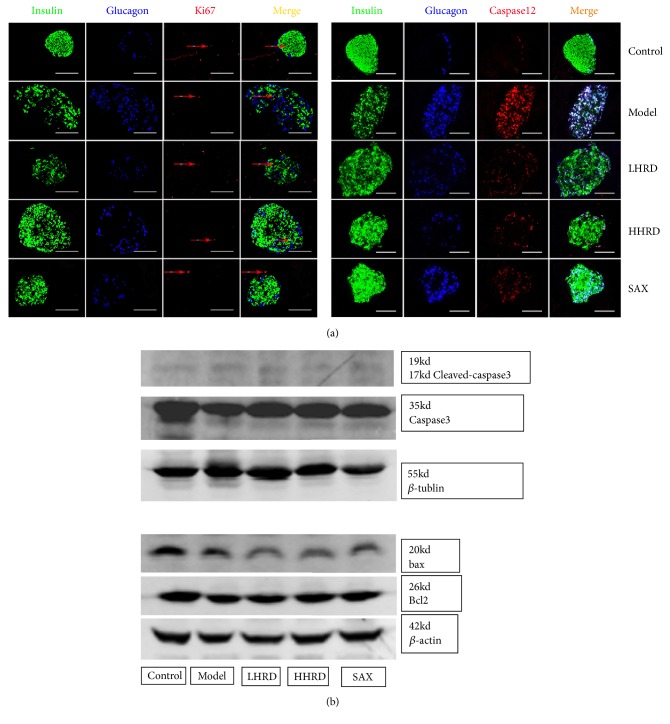
*Immunofluorescence images of ki67/insulin/glucagon and caspase 12/insulin/glucagon coexpression and Western blotting images of cleaved-caspase 3/caspase 3, bcl2/bax expression in pancreas.* (a) Immunofluorescence images showing glucagon (blue), insulin (green), and Ki67 (red) coexpression in the islets. (b) Immunofluorescence images showing glucagon (blue), insulin (green), and caspase 12 (red) coexpression in the islets. (c) Western blotting showing t cleaved-caspase 3/caspase 3, bcl2/bax expression in the pancreas. n=4-6 mice in each group. All immunofluorescence images were taken with a fluorescence microscope (×400) and Odyssey Scanning.

Compared with those of the control group (Figure 7), no difference was found in ki67, bcl2/bax and cleaved-caspase 3/caspase 3 expression among all of the groups. However, an increase of caspase 12 expression was found in the model group. After the intervention, caspase 12 expression was significantly decreased.

### 3.7. Effects of HRD on Cleaved-Notch1 and Ngn3 Expression in Islets

**Figure 8:**
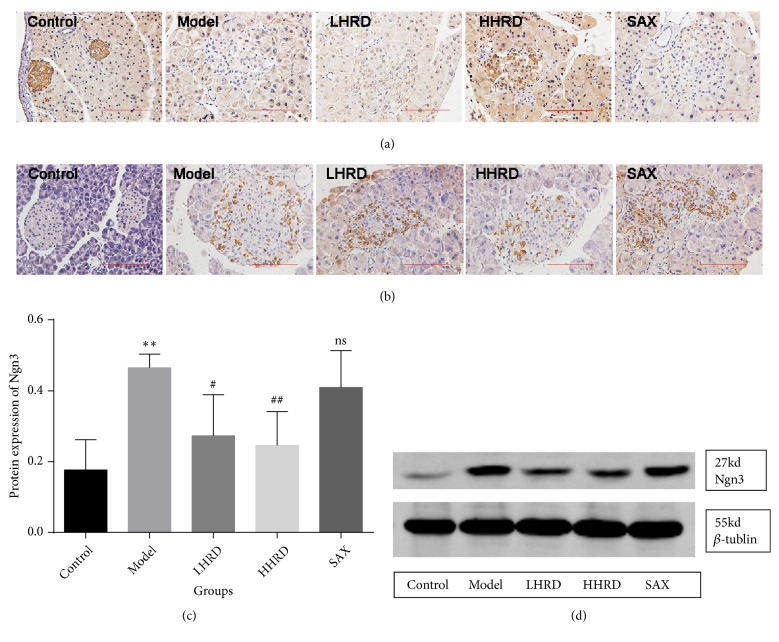
*Immunohistochemistry images for cleaved Notch1 and Ngn3.* (a) Representative immunohistochemistry images of cleaved Notch1 in the islets. (b) Representative immunohistochemistry images of Ngn3 in the islets. (c) and (d) show statistical analysis figures and Western blotting images. n=3-5 mice in each group. All IHC images were taken with an electronic microscope (×400).

The Notch1 signal pathway is closely related to differentiation of the pancreas [26]. Ngn3, which is a dedifferentiation marker, is a downstream signalling molecule of Notch1 [27]. Compared with that of the control group (Figure 8), cleaved Notch1, which is the activated form of Notch1, was decreased significantly in the model group. However, the Ngn3 level in pancreas was obviously increased. After the intervention, cleaved Notch1 expression was increased significantly in the HHRD group, whereas Ngn3 level obviously decreased.

## 4. Discussion

Evidence from existing studies showed that both Coptidis Rhizoma and Panax ginseng had extraordinary effects on decreasing hyperglycaemia [28–31]. The underlying mechanisms have been widely studied, especially for their bioactive components. For example, in addition to its antidiabetes action, berberine showed anticancer and antilipid effects [32, 33]. Moreover, the ginsenosides Rg1 and Rb1, which are key components of Panax ginseng, showed protective effects on cardiomyocytes and relieved heart failure [34, 35]. However, the precise mechanism involved in alleviating diabetes is unclear. In addition, few experiments have explored the effects of Coptidis Rhizoma and Panax ginseng combination. Consequently, studies of HRD with traditional combination are essential.

In general, our data were consistent with those of previous studies of decreases in glucose and increases in insulin concentration. Those data illustrated that HRD could not only ameliorate IR but could also improve insulin secretion. The increase in insulin was ascribed to inhibition of dipeptidyl peptidase-4 in the SAX group, whereas the cause of the increase in insulin in the HHRD group was unclear. Furthermore, we evaluated the pancreatic *β* cells (insulin^+^ cells) and *α* cells (glucagon^+^ cells) in islets using immunofluorescence. Interestingly, the *β* cell level was decreased and *α* cell level was increased significantly in the model group compared with those of the control group. A similar phenomenon was found in human diabetic pancreas (Supplementary Figure 2). The disturbance was partly attenuated after the intervention, especially in the HHRD group. The restoration of *β* cells probably explained why the serum insulin concentration increased significantly in the HHRD group. Certainly, the mechanism was explored further. A previous study illustrated that the *β* cell numbers were decreased significantly in the diabetic group due to the apoptosis [36]. Nevertheless, the current study showed that the failure of *β* cells should be attributed to their dedifferentiation rather than apoptosis [4, 37]. This disputation promoted us to examine the proliferative (ki67 and bcl2) and apoptotic proteins (bax, cleaved-caspase 3/caspase 3 and caspase 12) in the pancreas, respectively. Surprisingly, no difference was found in the expression of those proteins among groups except for caspase 12. Caspase 12 expression was increased significantly in the model group. In addition, its expression overlapped with glucagon rather than with insulin in the islets (Figure 7). According to a previous study, caspase 12 could not be activated by cell death or mitochondrial apoptotic factors but instead could only be activated by endoplasmic reticulum (ER) stress [38]. Moreover, Johnson et al. demonstrated that PDX1 could maintain the endoplasmic reticulum calcium concentration to repress ER-stress induced apoptosis through regulation of the sarcoendoplasmic reticulum calcium ATPase 2b (SERCA2b) in islet *β* cells [36]. This finding probably indicated that the HRD could lower the ER-stress-mediated apoptosis by increasing the PDX1 level in the islets.

The Notch signal pathway is important for regulating endocrine cells differentiation [39]. Additionally, it has an effect on restoration of dedifferentiated *β* cells [40]. Ngn3 is a basic helix-loop-helix (bHLH) protein and a progenitor cell marker in islets. A series of studies demonstrated that Notch1 could modulate Ngn3 expression. Murtaugh et al. showed that Notch1 could inhibit Nng3-expressing endocrine precursors but had no effect on differentiated endocrine cells [26]. In addition, one study favoured the hypothesis that Notch1 could repress pancreatic Ngn3 expression [41]. Consequently, we proposed the hypothesis that HRD reinstated the identity of endocrine cells by regulating the Notch1/Ngn3 signal pathway. We examined Notch1 and Nng3 expression with immunohistochemistry and Western blotting. Surprisingly, a significant decrease in Notch1 and increase in Ngn3 were observed in the model group compared with those of the control group. A similar tendency in Ngn3 expression appeared in the human pancreas (Supplementary Figure 3). After the intervention, Notch1 and Ngn3 expression was restored significantly in the HHRD group. These data demonstrated that the Notch1/Ngn3 signalling pathway was probably involved in *β* cell dedifferentiation.

Moreover, the increase in Ngn3 resulted from the dedifferentiation of pancreatic *β* cells in the islets and was probably attributed to the decrease in the *β* cell mass. However, the reason why the pancreatic *α* cells were increased significantly in the diabetic model group was unclear. We performed multicolour fluorescence analysis to explore the relationship between *β*and *α* cells. Our data showed that coexpression of insulin, glucagon, and PDX1 in islets was increased obviously in the model group. These data may favour a hypothesis that *β* cell transdifferentiation leads to a decrease in *β* cells and increase in *α* cells. After the intervention, *α* cell mass decreased and the *β* cell mass increased. Moreover, insulin, glucagon, and PDX1 coexpression was decreased significantly in the HHRD group. Those data may illustrate that HRD can prevent *β* cell transdifferentiation into *α* cell.

However, our study had some limitations. First, we did not detect insulin, glucagon, and Ngn3 coexpression in islets with immunofluorescence, which could present their locations directly. Second, whether the Notch1/Ngn3 signal pathway mediates the dedifferentiation needs further examination in vitro. Third, the mechanism of *β* cell transdifferentiation into *α* cell is far from clear. Last, the slight effect of saxagliptin on regulating glucose metabolism in db/db mice may due to the low daily dose.

## 5. Conclusions

In conclusion, our data favoured the hypothesis that HRD could improve glucose metabolism in db/db mice. HRD maintained the *β* cell identity by preventing dedifferentiation and transdifferentiation. This process probably involved the Notch1/Ngn3 signal pathway.

## Data Availability

The data used to support the findings of this study are available from the corresponding author upon request.

## Supplementary Materials

Supplementary Materials See Figures S1-S3 in the Supplementary Material for the comprehensive image analysis.* Supplementary Figure *1: body weight, 24-hour food intake and serum lipids levels. (a) Body weight in each week, mean±SD; (b) 24-hour food intake in each week, mean±SEM; (c) serum triglyceride concentration, mean±SD; (d) serum total cholesterol, mean±SD. n=5-10. *∗∗∗∗p* < 0.0001 versus. control group.* Supplementary Figure *2: non-T2DM and T2DM human pancreas. The top line of pictures showed the insulin (green) and glucagon (red) in non-T2DM human pancreas. The lower line of pictures showed the insulin (green) and glucagon (red) in T2DM human pancreas. n=3 in each group. T2DM, Type 2 Diabetic Mellitus.* Supplementary Figure *3: immunohistochemical images showed the expression of Ngn3 in Non-T2DM and T2DM patients. n=3 in each group. Ngn3, Neurogenin3; T2DM, Type 2 Diabetic Mellitus.
