# Supplementary material for: Effects of Huanglian-Renshen-Decoction, a Fixed Mixture of Traditional Chinese Medicine, on the Improvement of Glucose Metabolism by Maintenance of Pancreatic β Cell Identity in db/db Mice
Source: Evid Based Complement Alternat Med. 2019 Mar 19;2019:1232913. doi: 10.1155/2019/1232913 (PMC6444265; doi:10.1155/2019/1232913)
Supplement: Supplementary Materials — See Figures S1-S3 in the Supplementary Material for the comprehensive image analysis. Supplementary Figure 1: body weight, 24-hour food intake and serum lipids levels. (a) Body weight in each week, mean±SD; (b) 24-hour food intake in each week, mean±SEM; (c) serum triglyceride concentration, mean±SD; (d) serum total cholesterol, mean±SD. n=5-10. ∗∗∗∗p < 0.0001 versus. control group. Supplementary Figure 2: non-T2DM and T2DM human pancreas. The top line of pictures showed the insulin (green) and glucagon (red) in non-T2DM human pancreas. The lower line of pictures showed the insulin (green) and glucagon (red) in T2DM human pancreas. n=3 in each group. T2DM, Type 2 Diabetic Mellitus. Supplementary Figure 3: immunohistochemical images showed the expression of Ngn3 in Non-T2DM and T2DM patients. n=3 in each group. Ngn3, Neurogenin3; T2DM, Type 2 Diabetic Mellitus. [file 1232913.f1.zip › 1232913.f1.pptx]

## Slide 1
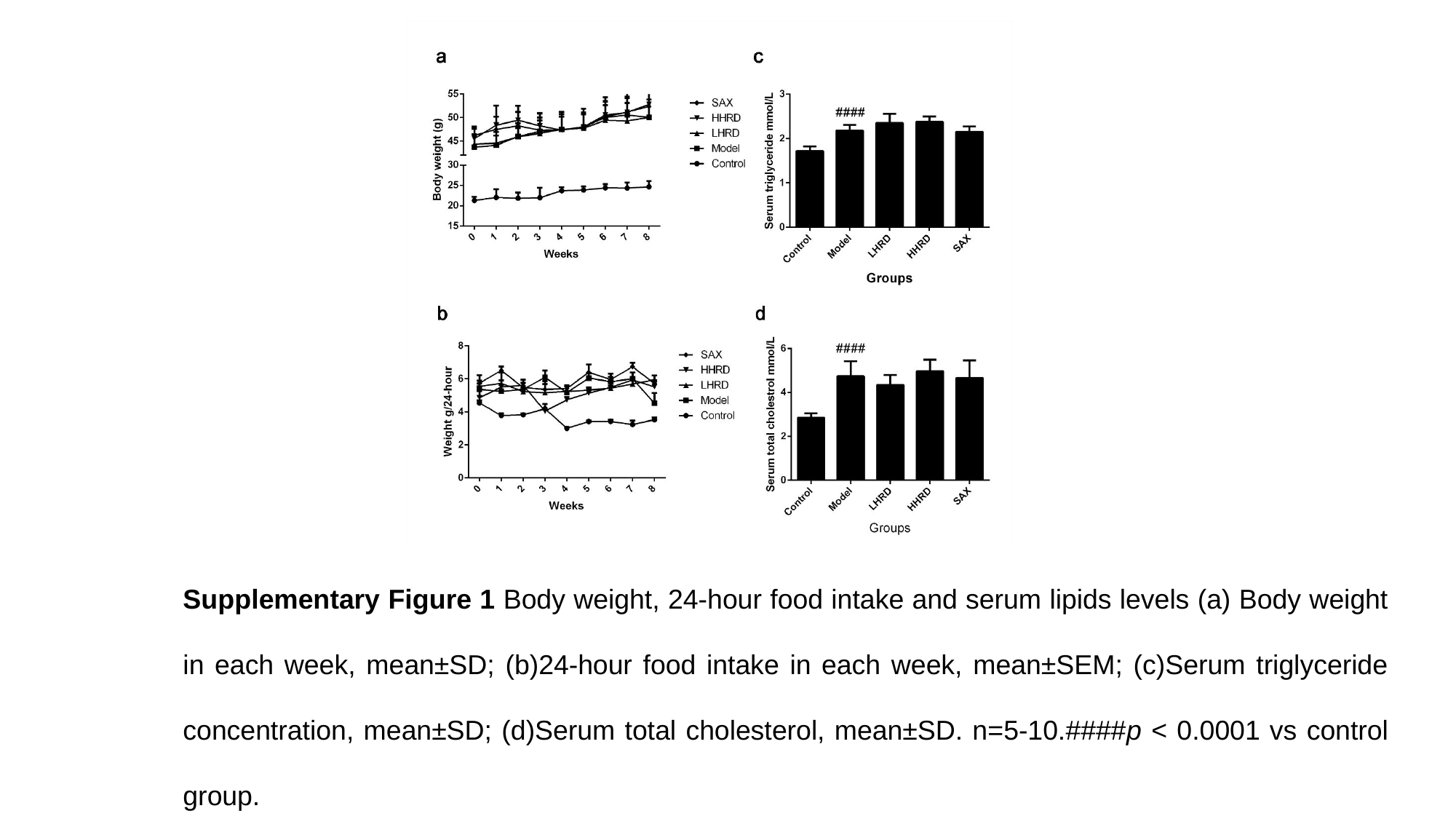

Supplementary Figure 1 Body weight, 24-hour food intake and serum lipids levels (a) Body weight in each week, mean±SD; (b)24-hour food intake in each week, mean±SEM; (c)Serum triglyceride concentration, mean±SD; (d)Serum total cholesterol, mean±SD. n=5-10.####p < 0.0001 vs control group.
